# The Influences of Emotion on Learning and Memory

**DOI:** 10.3389/fpsyg.2017.01454

**Published:** 2017-08-24

**Authors:** Chai M. Tyng, Hafeez U. Amin, Mohamad N. M. Saad, Aamir S. Malik

**Affiliations:** Centre for Intelligent Signal and Imaging Research (CISIR), Department of Electrical and Electronic Engineering, Universiti Teknologi Petronas Seri Iskandar, Malaysia

**Keywords:** emotional valence, arousal, learning, memory, prefrontal cortex (PFC), medial temporal lobe (MTL), amygdala, neuroimaging

## Abstract

Emotion has a substantial influence on the cognitive processes in humans, including perception, attention, learning, memory, reasoning, and problem solving. Emotion has a particularly strong influence on attention, especially modulating the selectivity of attention as well as motivating action and behavior. This attentional and executive control is intimately linked to learning processes, as intrinsically limited attentional capacities are better focused on relevant information. Emotion also facilitates encoding and helps retrieval of information efficiently. However, the effects of emotion on learning and memory are not always univalent, as studies have reported that emotion either enhances or impairs learning and long-term memory (LTM) retention, depending on a range of factors. Recent neuroimaging findings have indicated that the amygdala and prefrontal cortex cooperate with the medial temporal lobe in an integrated manner that affords (i) the amygdala modulating memory consolidation; (ii) the prefrontal cortex mediating memory encoding and formation; and (iii) the hippocampus for successful learning and LTM retention. We also review the nested hierarchies of circular emotional control and cognitive regulation (bottom-up and top-down influences) within the brain to achieve optimal integration of emotional and cognitive processing. This review highlights a basic evolutionary approach to emotion to understand the effects of emotion on learning and memory and the functional roles played by various brain regions and their mutual interactions in relation to emotional processing. We also summarize the current state of knowledge on the impact of emotion on memory and map implications for educational settings. In addition to elucidating the memory-enhancing effects of emotion, neuroimaging findings extend our understanding of emotional influences on learning and memory processes; this knowledge may be useful for the design of effective educational curricula to provide a conducive learning environment for both traditional “live” learning in classrooms and “virtual” learning through online-based educational technologies.

## Introduction

Emotional experiences are ubiquitous in nature and important and perhaps even critical in academic settings, as emotion modulates virtually every aspect of cognition. Tests, examinations, homework, and deadlines are associated with different emotional states that encompass frustration, anxiety, and boredom. Even subject matter influences emotions that affect one’s ability to learn and remember. The usage of computer-based multimedia educational technologies, such as intelligent tutoring systems (ITSs) and massive open online courses (MOOCs), which are gradually replacing traditional face-to-face learning environments, is increasing. This may induce various emotional experiences in learners. Hence, emotional influences should be carefully considered in educational courses design to maximize learner engagement as well as improve learning and long-term retention of the material (Shen et al., 2009). Numerous studies have reported that human cognitive processes are affected by emotions, including attention (Vuilleumier, 2005), learning and memory (Phelps, 2004; Um et al., 2012), reasoning (Jung et al., 2014), and problem-solving (Isen et al., 1987). These factors are critical in educational domains because when students face such difficulties, it defeats the purpose of schooling and can potentially render it meaningless. Most importantly, emotional stimuli appear to consume more attentional resources than non-emotional stimuli (Schupp et al., 2007). Moreover, attentional and motivational components of emotion have been linked to heightened learning and memory (Pekrun, 1992; Seli et al., 2016). Hence, emotional experiences/stimuli appear to be remembered vividly and accurately, with great resilience over time.

Recent studies using functional neuroimaging techniques detect and recognize human emotional states and have become a topic of increasing research in cognitive neuroscience, affective neuroscience, and educational psychology to optimize learning and memory outcomes (Carew and Magsamen, 2010; Um et al., 2012). Human emotions comprise complex interactions of subjective feelings as well as physiological and behavioral responses that are especially triggered by external stimuli, which are subjectively perceived as “personally significant.” Three different approaches are used to monitor the changes in emotional states: (1) *subjective approaches* that assess subjective feelings and experiences; (2) *behavioral investigations* of facial expressions (Jack and Schyns, 2015), vocal expressions (Russell et al., 2003), and gestural changes (Dael et al., 2012); and (3) *objective approaches* via physiological responses that include electrical and hemodynamic of the central nervous system (CNS) activities (Vytal and Hamann, 2010) in addition to autonomic nervous system (ANS) responses such as heart rate, respiratory volume/rate, skin temperature, skin conductance and blood volume pulses (Li and Chen, 2006). The CNS and ANS physiological responses (brain vs. body organs) can be objectively measured via neuroimaging and biosensors and are more difficult to consciously conceal or manipulate compared to subjective and behavioral responses. Although functional neuroimaging enables us to identify brain regions of interest for cognitive and emotional processing, it is difficult to comprehend emotional influences on learning and memory retrieval without a fundamental understanding of the brain’s inherent emotional operating systems.

The aim of this current article was to highlight an evolutionary approach to emotion, which may facilitate understanding of the effects of emotion on learning and memory. We initially present the terminology used in affective neuroscience studies, describe the roles of emotion and motivation in learning and memory, and outline the evolutionary framework and the seven primary emotional system. This is followed by the emotional-cognitive interactions in the various brain regions that are intimately involved in emotion and memory systems. This is performed to define the congruent interactions in these regions are associated with long-term memory (LTM) retention. We then discuss the emerging studies that further our understanding of emotional effects deriving from different modalities of emotional content. This is followed by a discussion of four major functional neuroimaging techniques, including functional magnetic resonance imaging (fMRI), positron emission tomography (PET), electroencephalography (EEG), and functional near-infrared spectroscopy (fNIRS). We then present the important factors for consideration in experimental design, followed by a description of psychiatric disorders, such as depression and anxiety, which are emotionally charged dysfunctions that are strongly detrimental to cognitive performance. Our review ends with concluding remarks on the current issues and future research possibilities with respect to the efficient enhancement of educational practices and technologies.

## Emotions, Moods, Feelings, Affects and Drives

Subjective terms used in affective neuroscience include emotions, moods, feelings, affects and drives. Although emotion has long been studied, it bears no single definition. A review of 92 putative definitions and nine skeptical statements (Kleinginna and Kleinginna, 1981) suggests a definition with a rather broad consensus:

Emotions describe a complex set of interactions between subjective and objective variables that are mediated by neural and hormonal systems, which can (a) give rise to affective experiences of emotional valence (pleasure-displeasure) and emotional arousal (high-low activation/calming-arousing); (b) generate cognitive processes such as emotionally relevant perceptual affect, appraisals, labeling processes; (c) activate widespread psychological and physiological changes to the arousing conditions; and (d) motivate behavior that is often but not always expressive, goal-directed and adaptive.

Although this definition may be adequate for everyday purposes, it does not encompass some important aspects of emotional systems such as how emotions operate to create subjectively experienced feelings and how they control personality dimensions. Accordingly, Panksepp (1998) suggested the following:

Emotions are the psychoneural processes that are influential in controlling the vigor and patterning of actions in the dynamic flow of intense behavioral interchanges between animals as well as with certain objects that are important for survival. Hence, each emotion has a characteristic “feeling tone” that is especially important in encoding the intrinsic values of these interactions, depending on their likelihood of either promoting or hindering survival (both in the immediate “personal” and long-term “reproductive” sense). Subjective experiential-feelings arise from the interactions of various emotional systems with the fundamental brain substrates of “the self,” that is important in encoding new information as well as retrieving information on subsequent events and allowing individuals efficiently to generalize new events and make decisions.

He went further to propose seven primary emotional systems/prototype emotional states, namely SEEKING, RAGE, FEAR, LUST, CARE, PANIC/GRIEF, and PLAY that represent basic foundations for living and learning.

Moods last longer than emotions, which are also characterized by positive and negative moods. In contrast, feelings refer to mental experiences that are necessarily valence, either good or bad as well as *accompanied* by internal physiological changes in the body, specifically the viscera, including the heart, lungs, and gut, for maintaining or restoring homeostatic balances. Feelings are not commonly caused emotions. Because the generation of emotional feelings requires a neural re-mapping of different features of the body state in the CNS, resulting from cognitive “appraisal” where the anterior insular cortex plays a key integrative role (Craig and Craig, 2009; Damasio and Carvalho, 2013). Nonetheless, Panksepp (2005) has defended the view that emotional operating systems (caudal and medial subcortical brain regions) appeared to generate emotional experiences via *localized electrical stimulation of the brain stimulation* (ESB) rather dependent on changes of the external environment or bodily states. Affects are subjective experienced emotional feelings that are difficult to describe, but have been linked to bodily states such as homeostatic drives (hunger and thirst) and external stimuli (visual, auditory, taste, touch, smell) (Panksepp, 2005). The latter are sometimes called “core affect,” which refers to consciously accessible elemental processes involving pleasure and arousal that span bipolar dimensions (Russell and Barrett, 1999). In addition, a “drive” is an inherent action program that is responsible for the satisfaction of basic and instinctual (biologically pre-set) physiological needs, e.g., hunger, thirst, libido, exploration, play, and attachment to mates (Panksepp, 1998); this is sometimes called “homeostatic drive.” In brief, a crucial characteristic shared by emotion, mood, feeling, affect and drive is their intrinsic valence, which lies on the spectrum of positive and negative valence (pleasure-displeasure/goodness-badness). The term *emotion* exemplifies the “umbrella” concept that includes affective, cognitive, behavioral, expressive and physiological changes; emotion is triggered by external stimuli and associated with the combination of feeling and motivation.

## Recent Evidence Regarding the Role of Emotion in Learning and Memory

The impact of emotion on learning processes is the focus of many current studies. Although it is well established that emotions influence memory retention and recall, in terms of learning, the question of emotional impacts remains questionable. Some studies report that positive emotions facilitate learning and contribute to academic achievement, being mediated by the levels of self-motivation and satisfaction with learning materials (Um et al., 2012). Conversely, a recent study reported that negative learning-centered state (confusion) improve learning because of an increased focus of attention on learning material that leads to higher performances on post tests and transfer tests (D’Mello et al., 2014). Confusion is not an emotion but a cognitive disequilibrium state induced by contradictory data. A confused student might be frustrated with their poor understanding of subject matter, and this is related to both the SEEKING and RAGE systems, with a low-level of activation of rage or irritation, and amplification of SEEKING. Hence, motivated students who respond to their confusion seek new understanding by doing additional cognitive work. Further clarification of this enhances learning. Moreover, stress, a negative emotional state, has also been reported to facilitate and/or impair both learning and memory, depending on intensity and duration (Vogel and Schwabe, 2016). More specifically, mild and acute stress facilitates learning and cognitive performance, while excess and chronic stress impairs learning and is detrimental to memory performance. Many other negative consequences attend owing to *overactivity* of the hypothalamic-pituitary-adrenal (HPA) axis, which results in both impaired synaptic plasticity and learning ability (Joëls et al., 2004). Nonetheless, confounding influences of emotions on learning and memory can be explained in terms of attentional and motivational components. Attentional components enhance perceptual processing, which then helps to select and organize salient information via a “bottom-up” approach to higher brain functions and awareness (Vuilleumier, 2005). Motivational components induce curiosity, which is a state associated with psychological interest in novel and/or surprising activities (stimuli). A curiosity state encourages further exploration and apparently prepares the brain to learn and remember in both children and adults (Oudeyer et al., 2016). The term “surprising” might be conceptualized as an *incongruous* situation (expectancy violation) refers to a discrepancy between prior expectations and the new information; it may drive a cognitive reset for “learned content” that draws one’s attention.

Similarly, emotionally enhanced memory functions have been reported in relation to selective attention elicited by emotionally salient stimuli (Vuilleumier, 2005; Schupp et al., 2007). During the initial perceptual stage, attention is biased toward emotionally salient information that supports detection by the salient input. Thus, stimulating selective attention increases the likelihood for emotional information to become encoded in LTM storage associated with a top-down control in sensory pathways that are modulated by the frontal and parietal cortices. This is an example of an indirect influence on perception and attention that regulates selective sensory processing and behavioral determination (Vuilleumier, 2005). Because the human sensory systems have no capacity to simultaneously process everything at once, which necessitates attentional mechanisms. Top-down attentional processing obtains adequate attentional resource allocation to process emotional valence information for encoding and retrieval via cooperation with the brain regions such as the ventromedial prefrontal cortex and superior temporal sulcus, along with the primary visual cortex (helps to realize both emotion and conceptualization). Similarly, experimental studies have examined the phenomenon by using various attentional tasks, including filtering (dichotic listening and Stroop task), search (visual search), cuing (attentional probe, spatial cuing) and attentional blink [rapid serial visual presentation (RSVP)] paradigms (Yiend, 2010). These investigations demonstrated biased attentional processing toward emotionally stimulating material content attended by increased sensory responses. One study reported that emotional stimuli induce a “pop-out” effect that leads to the attentional capture and privileged processing (Öhman et al., 2001). Moreover, a study using the RSVP paradigm compared healthy subjects with a group of patients with bilateral amygdala damage. The results revealed that healthy subjects exhibited increased perception and attention toward emotional words compared to patients, indicating that the amygdala plays a crucial role in emotional processing (Anderson and Phelps, 2001). In addition, functional neuroimaging showed that the insular cortex, the secondary somatosensory cortex, the cingulate cortex and nuclei in the tegmentum and hypothalamus are the brain regions that regulate attentional focus by integrating external and internal inputs to create emotional feeling states, thus modulating a motivational state that obtains homeostasis (Damasio et al., 2000). All emotional systems associated with strong motivational components such as psychological salient bodily need states operate through the SEEKING system that motivates appetitive/exploratory behavior to acquire resources needed for survival (Montag and Panksepp, 2017).

The distinction between emotion and homeostasis, is the process of regulation for continuously changing internal states via appropriate corrective responses that respond to both internal and external environmental conditions to maintain an optimal physiological state in the body. *Homeostatic affects*, such as hunger and thirst, are not considered prototype emotional states. Because homeostatic affects have never been mapped using ESB that arouse basic emotional responses (Panksepp, 2005, 2007). However, emotional prototypes can be thought of as evolutionary *extensions/predictions* of impending homeostatic threats; for example, SEEKING might be an evolutionary extension of intense hunger and thirst (the major sources of suffering that signal energy depletion to search for food and water intake) (Watt, 2012). Homeostatic imbalances engage the mesolimbic motivational system via hypothalamic interactions with the extended trajectory of the SEEKING system [centrally including the lateral hypothalamus, ventral basal ganglia, and ventral tegmental area (VTA)]. It is the distributed functional network that serves the general function of finding resources for survival that gets hungry animals to food, thirsty animals to water, cold animals to warmer environments, etc. (Panksepp, 1998). To summarize, both emotion and motivation are crucial for the maintenance of psychological and physiological homeostasis, while emotional roles are particularly important in the process of encoding new information containing emotional components. The latter increases attention toward salient new information by selectively enhancing detection, evaluation, and extraction of data for memorization. In addition, motivational components promote learning and enhance subsequent memory retrieval while generalizing new events consequent to adaptive physiological changes.

## The Evolutionary Framework of Emotion and The Seven Primary Emotional Systems

**FIGURE 1:**
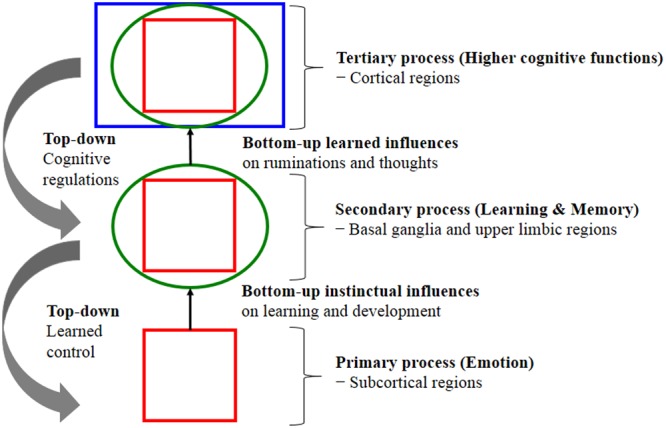
Shows the nested hierarchies of circular emotional control and cognitive regulation for “bottom-up” influences and “top-down” regulations. The schematic shows conceptual relationships between primary processes of emotional system (lower brain function), as well as secondary processes of cognitive system and tertiary processing (higher brain function). Primary emotional processing for homeostatic, sensory and emotional affects facilitate secondary learning and memory processing via the “SEEKING” system that promotes survival and reproductive success (bottom-up instinctual influences). As secondary processes are continually integrated with primary emotional processing, they mature to higher brain cognitive faculties to generate effective solutions for living and subsequently exert top-down regulatory control over behavior. The primary emotional processing is mediated by complex unconditioned emotional responses (evolutionary “memories”) through “Law of Affect”; sometimes called “reinforcement principle” that explains how the brain emotional networks control learning. This bi-circular causation for higher brain functionality is coordinated by lower brain functions [adapted from (Panksepp and Solms, 2012)].

Evolution built our higher minds (the faculty of consciousness and thoughts) on a foundation of primary-process of emotional mechanism that preprogrammed executive action systems (the prototype emotions) rely on cognitive processing (interpretation) and appraisal in the organisms attempt to decipher the type of situation they might be in; in other words, how to deal with emotionally challenging situations, whether it is a *play* situation or a *threat* situation (where RAGE and FEAR might be the appropriate system to recruit). Emotion offers preprogrammed but partially modifiable (under the secondary process of learning and memory) behavioral routines in the service of the solution of prototypical adaptive challenges, particularly in dealing with friend vs. foe; these routines are evolutionary *extensions* of homeostasis and embed a prediction beyond the current situation to a potentially future homeostatic benefit or threat. Thus, evolution uses whatever sources for survival and procreative success. According to Panksepp and Solms (2012), key CNS emotional-affective processes are (1) Primary-process emotions; (2) Secondary-process learning and memory; and (3) Tertiary-process higher cognitive functions. Fundamentally, primary emotional processes regulate unconditioned emotional actions that anticipate survival needs and consequently guide secondary process via associative learning mechanisms (classical/Pavlovian and instrumental/operant conditioning). Subsequently, learning process sends relevant information to higher brain regions such as the prefrontal cortex to perform tertiary cognition process that allows planning for future based on past experiences, stored in LTM. In other words, the brain’s neurodevelopment trajectory and “wiring up” activations show that there is a genetically coded aversion to situations that generate RAGE, FEAR and other negative states for minimizing painful things and maximizing pleasurable kinds of stimulation. These are not learned-*all* learning (secondary-process) is piggybacked on top of the “primary-process emotions” that are governed by “Law of Affect” (see **Figure 1**). What now follows is an explanation of these CNS emotional-affective processing sub-levels and their inter-relationships.

### Primary-Process Emotions (Prototype Emotional States)

The emotional operating system is an inherited and genetically encoded circuitry that anticipates key survival and homeostatic needs. Thus, animals and humans share primary emotional network at the subcortical level, which includes the midbrain’s periaqueductal grey (PAG) and VTA, basal ganglia (amygdala and nucleus accumbens), and insula, as well as diencephalon (the cingulate and medial frontal cortices through the lateral and medial hypothalamus and medial thalamus). Subcortical brain regions are involved in three sub-components of affects: (1) core emotional feelings (fear, anger, joy and various forms of distress); (2) homeostatic drives/motivational experiences (hunger and thirst); and (3) sensory affects (pain, taste, temperature and disgust). Primary-process emotions are not unconscious. Strong emotion is intrinsically conscious at least in the sense that it is experienced even if we might mislabel it, or animal clearly is not able to attach a semantic label-these are simply not realistic standards for determining whether something is conscious or not conscious. Nonetheless, the emotional experiences guide behavior to promote survival and procreative success as well as mediate learning (‘*rewarding*’ and ‘*punishing*’ learning effects) and thinking at secondary and tertiary levels.

### Secondary-Process Emotions (Learning and Memory)

Primary emotional systems guide associative learning and memory (classical/operant conditioning and emotional habit) processes via the mediation of emotional networks. This includes the basal ganglia (basolateral and central amygdala, nucleus accumbens, thalamus and dorsal striatum), and the medial temporal lobe (MTL) including hippocampus as well as the entorhinal cortex, perirhinal cortex, and parahippocampal cortices that responsible for declarative memories. Thus, secondary processes of learning and memory scrutinize and regulate emotional feelings in relation to environmental events that subsequently refine effective solutions to living.

### Tertiary-Process Emotions (Higher Cognitive Functions)

Higher cognitive functions operate within the cortical regions, including the frontal cortex for awareness and consciousness functions such as thinking, planning, emotional regulation and free-will (intention-to-act), which mediate emotional feelings. Hence, cognition is an extension of emotion (just as emotion is an extension of homeostasis aforementioned). Tertiary processes are continually integrated with the secondary processes and reach a mature level (higher brain functions) to better anticipating key survival issues, thus yielding cognitive control of emotion via “top-down” regulation. In other words, brain-mind evolution enables human to reason but also regulate our emotions.

**FIGURE 2:**
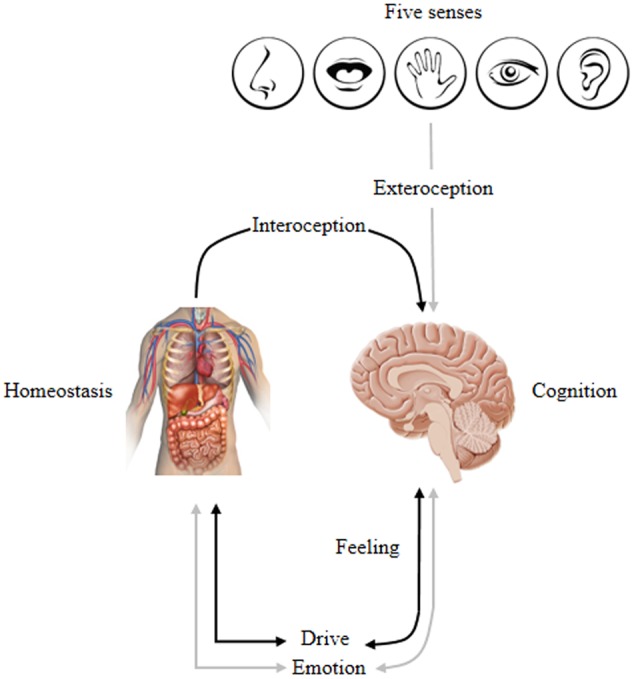
Conceptually maps the homeostatic regulation of internal and external inputs that affect cognition, emotion, feeling, and drive: Inputs → Homeostasis ↔ Emotion^∗^ ↔ Cognition. This lead to the experience of one’s self via overt behavior that is biased by a specific emotion stimulated by bodily changes that underlie psychological/physiological states. ^∗^Represents emotion associated with a combination of feeling and motivation/drive; ↔ indicates a bi-directional interaction; and → indicates a one-directional relationship. Adapted from Damasio and Carvalho (2013).

Psychologist Neisser (1963) suggested that cognition serves emotion and homeostatic needs where environmental information is evaluated in terms of its ability to satisfy or frustrate needs. In other words, cognition is in the service of satisfying emotional and homeostatic needs. This infers that cognition modulates, activates and inhibits emotion. Hence, emotion is not a simple linear event but rather a feedback process that autonomously restores an individual’s state of equilibrium. More specifically stated, emotion regulates the allocation of processing resources and determines our behavior by tuning us to the world in certain biased ways, thus steering us toward things that “feel good” while avoiding things that “feel bad.” This indicates that emotion guides and motivates cognition that promotes survival by guiding behavior and desires according to unique goal orientation (Northoff et al., 2006). Therefore, the CNS maintains complex processes by continually monitoring internal and external environments. For example, changes in internal environments (contraction of visceral muscles, heart rate, etc.) are sensed by an interoceptive system (afferent peripheral nerves) that signals the sensory cortex (primary, secondary and somatosensory) for integration and processing. Thus, from an evolutionary perspective, human mental activity is driven by the ancient emotional and motivational brain systems shared by cross-mammalians that encode life-sustaining and life-detracting features to promote adaptive instinctual responses. Moreover, emotional and homeostasis mechanisms are characterized by intrinsic valence processing that is either a positive/pleasure or negative/displeasure bias. Homeostasis imbalance is universally experienced as negative emotional feelings and only becomes positively valenced when rectified. Hence, individuals sustain bodily changes that underlie psychological (emotional) and biological (homeostatic) influences on two sides, i.e., one side is oriented toward the survival and reproductive success that is associated with positively valenced emotional and physiologic homeostasis (anticipatory response) and the other responds to survival and reproductive failure associated with negatively valenced emotional and physiologic homeostasis (reactive response). Consequently, cognition modulates both emotional and homeostatic states by enhancing survival and maximizing rewards while minimizing risk and punishments. Thus, this evolutionary consideration suggests the brain as a ‘predictive engine’ to make it adaptive in a particular environment. **Figure 2** demonstrates this cyclic homeostatic regulation.

Panksepp (1998) identified seven primary emotional systems that govern mammalian brains as follows: SEEKING, RAGE, FEAR, LUST, CARE, PANIC/GRIEF, and PLAY. Here, we use UPPERCASE letters to denote unconditional emotional responses (emotional primes). These primary emotional neural networks are situated in the subcortical regions; moreover, the evidence demonstrates that decortication leaves primary emotional systems intact (Panksepp et al., 1994). Hence, cortical regions are non-essential for the generation of prototype emotional states but are responsible for their modulation and regulation. The present article emphasizes SEEKING because it is the most fundamental of the primary emotional systems and is crucial for learning and memory. The SEEKING system facilitates learning because when fully aroused, it fills the mind with interest that then motivates the individual to search out and learn things that they need, crave and desire. Accordingly, SEEKING generates and sustains curiosity’s engagement for a particular purpose while also promoting learning via its mediation of anticipatory eagerness (Oudeyer et al., 2016). In other words, the SEEKING system has been designed to automatically learn by exploring anything that results in acquired behavioral manifestations for survival operations, all the way from the mesolimbic-mesocortical dopamine system through to the prefrontal cortex (PFC); thus, it is intimately linked with LTM formation (Blumenfeld and Ranganath, 2007). Consequently, it is the foundation of secondary learning and higher cognitive processes when compared with the remaining six emotional systems. However, this system is less activated during chronic stress, sickness, and depression, all of which are likely to impair learning and various higher cognitions. On the other hand, *overactivity* of this system promotes excessively impulsive behaviors attended by manic thoughts and psychotic delusions. Moreover, massive lesion of SEEKING’s neural network (midline subcortical regions-the PAG, VTA, nucleus accumbens (NAc), medial forebrain and anterior cingulate) lead to consciousness disorder, specifically *akinetic mutism* (AKM) syndrome that the patient appears wakeful, attentive but motionless (Schiff and Plum, 2000; Watt and Pincus, 2004). In brief, the SEEKING system holds a critical position that optimizes the performance of emotion, motivation, and cognition processes by generating positive subjective emotional states-positive expectancy, enthusiastic exploration, and hopefulness. Because the seven primary emotional systems and their associated key neuroanatomical and key neurochemical features have been reviewed elsewhere (Panksepp, 2011a,b), they are not covered in this review.

## Emotion–Cognition Interactions and its Impacts on Learning and Memory

Studies in psychology (Metcalfe and Mischel, 1999) and neuroscience (Dolcos et al., 2011) proposed that cognition and emotion processes are operated at two separate but interacting systems: (i) the “cool cognitive system” is hippocampus-based that is associated with emotionally neutral cognitive functions as well as cognitive controls; and (ii) the “hot emotional system” is amygdala-based that responsible for emotional processing and responses toward unconditioned emotional stimuli such as appetitive and fear-evoking conditions. In addition, an early view of a dorsal/ventral stream distinction was commonly reported between both systems. The dorsal stream encompasses the dorsolateral prefrontal cortex (DLPFC) and lateral parietal cortex, which are involved in the cool system for active maintenance of controlled processes such as cognitive performance and the pursuit of goal-relevant information in working memory (WM) amidst interference. In contrast, the hot system involves the ventral neural system, including the amygdala, ventrolateral prefrontal cortex (VLPFC) and medial prefrontal cortex (mPFC) as well as orbitofrontal (OFC) and occipito-temporal cortex (OTC), all of which encompass emotional processing systems (Dolcos et al., 2011). Nonetheless, recent investigations claim that distinct cognitive and emotional neural systems are not separated but are deeply integrated and contain evidence of mediation and modulation (Dolcos et al., 2011; Okon-Singer et al., 2015). Consequently, emotions are now thought to influence the formation of a hippocampal-dependent memory system (Pessoa, 2008), exerting a long-term impact on learning and memory. In other words, although cognitive and affective processes can be independently conceptualized, it is not surprising that emotions powerfully modify cognitive appraisals and memory processes and vice versa. The innate emotional systems interact with higher brain systems and probably no an emotional state that is free of cognitive ramifications. If cortical functions were evolutionarily built upon the pre-existing subcortical foundations, it provides behavioral flexibility (Panksepp, 1998).

The hippocampus is located in the MTL and is thought to be responsible for the potentiation and consolidation of declarative memory before newly formed memories are distributed and stored in cortical regions (Squire, 1992). Moreover, evidence indicates that the hippocampus functions as a hub for brain network communications-a type of continuous exchange of information center that establishes LTM dominated by theta wave oscillations (Battaglia et al., 2011) that are correlated with learning and memory (Rutishauser et al., 2010). In other words, hippocampus plays a crucial role in hippocampal-dependent learning and declarative memories. Numerous studies have reported that the amygdala and hippocampus are synergistically activated during memory encoding to form a LTM of emotional information, that is associated with better retention (McGaugh et al., 1996; Richter-Levin and Akirav, 2000; Richardson et al., 2004). More importantly, these studies (fear-related learning) strongly suggest that the amygdala’s involvement in emotional processing strengthens the memory network by modulating memory consolidation; thus, emotional content is remembered better than neutral content.

In addition to amygdala-hippocampus interactions, one study reported that the PFC participates in emotional valence (pleasant vs. unpleasant) processing during WM (Perlstein et al., 2002). Simons and Spiers (2003) also reviewed studies of interactions between the PFC and MTL during the memory encoding and retrieval processes underlying successful LTM. They demonstrated that the PFC is crucial for LTM because it engages with the active maintenance of information linked to the cognitive control of selection, engagement, monitoring, and inhibition. Hence, it detects relevant data that appears worthwhile, which is then referred for encoding, thus leading to successful LTM (Simons and Spiers, 2003). Consistent findings were reported for recognition tasks investigated by fMRI where the left PFC-hippocampal network appeared to support successful memory encoding for neutral and negative non-arousing words. Simultaneously, amygdala-hippocampus activation was observed during the memory encoding of negative arousing words (Kensinger and Corkin, 2004). Moreover, Mega et al. (1996) proposed two divisions for the limbic system: (i) the paleocortex division (the amygdala, orbitofrontal cortex, temporal polar and anterior insula), and (ii) the archicortical division (the hippocampus and anterior cingulate cortex). The first component is responsible for the implicit integration of affects, drives and object associations; the second deals with explicit sensory processing, encoding, and attentional control. Although divided into two sub-divisions, the paleocortex and archicortical cortex remain integrated during learning. Here, the paleocortex appears to manage the internal environment for implicit learning while integrating affects, drives, and emotions. Simultaneously, the archicortical division appears to manage external environment input for explicit learning by facilitating attention selection with attendant implicit encoding. To some extent, the paleocortex system might come to exercise a supervisory role and link the ancient affective systems to the newer cognitive systems.

### Amygdala–Hippocampus Interactions

The findings of previous studies suggest that the amygdala is involved in emotional arousal processing and modulation of the memory processes (encoding and storage) that contribute to the emotional enhancement of memory (McGaugh et al., 1996; Richter-Levin and Akirav, 2000). Activation of the amygdala during the encoding of emotionally arousing information (both pleasant/unpleasant) has been reported that correlates with subsequent recall. Because of the interaction between *basolateral complex of the amygdala* (BLA) with other brain regions that are involved in consolidating memories, including the hippocampus, caudate nucleus, NAc, and other cortical regions. Thus, BLA activation results from emotionally arousing events, which appear to modulate memory storage-related regions that influence long-term memories (McGaugh, 2004). Memory consolidation is a part of the encoding and retention processes where labile memories of newly learned information become stabilized and are strengthened to form long-lasting memories (McGaugh, 2000). Moreover, the amygdala transmits direct feedback/projection along the entire rostral-caudal cortices to the visual cortex of the ventral stream system, including primary visual (V1) and temporal cortices (Amaral et al., 2003); furthermore, the amygdala activates the frontal and parietal regions during negative emotion processing that are involved in attention control. Consequently, during emotional processing, direct projections from the amygdala to sensory cortices enhance attentional mechanism might also allow the parallel processing of the attentional (fronto-parietal) system (Vuilleumier, 2005). This suggests that amygdala activation is associated with enhanced attention and is a part of how salience enhances information retention.

In addition to attentional biases toward emotional content during memory encoding, emotionally arousing experiences have been found to induce the release of adrenal stress hormones, followed by the activation of β-noradrenergic receptors in the BLA, which then release epinephrine and glucocorticoids in the BLA, while enhancing memory consolidation of emotional experiences (McGaugh and Roozendaal, 2002). Thus, there is evidence that the consolidation of new memory that is stimulated by emotionally arousing experiences can be enhanced through the modulating effects of the release of stress hormones and stress-activated neurotransmitters associated with amygdala activation. The BLA comprises the basal amygdala (BA) and lateral amygdala (LA), which project to numerous brain regions involved in learning and memory, including the hippocampus and PFC (Cahill and McGaugh, 1998; Sharot and Phelps, 2004; McGaugh, 2006). However, stress and emotion do not always induce strong memories of new information. Indeed, they have also been reported to inhibit WM and LTM under certain conditions related to mood and chronic stress (Schwabe and Wolf, 2010). Consequently, understanding, managing, and regulating emotion is critical to the development of enhanced learning programs informed by the significant impacts of learning and memory under different types of stress (Vogel and Schwabe, 2016).

### Prefrontal Cortex–Hippocampus Interaction

The PFC is located in the foremost anterior region of the frontal lobe and is associated with higher-order cognitive functions such as prediction and planning of/for the future (Barbey et al., 2009). Moreover, it is thought to act as a control center for selective attention (Squire et al., 2013), and also plays a critical role in WM as well as semantic processing, cognitive control, problem-solving, reasoning and emotional processing (Miller and Cohen, 2001; Yamasaki et al., 2002). The PFC is connected to sub-cortical regions in the limbic system, including the amygdala and various parts of the MTL (Simons and Spiers, 2003). Its involvement in WM and emotional processing are intimately connected with the MTL structures that decisively affect LTM encoding and retrieval (Blumenfeld and Ranganath, 2007) in addition to self-referential processing (Northoff et al., 2006). Structurally, the PFC is divided into five sub-regions: anterior (BA 10), dorsolateral (BA 9 and 46), ventrolateral (BA 44, 45, and 47), medial (BA 25 and 32) and orbitofrontal (BA 11, 12, and 14) (Simons and Spiers, 2003).

The mPFC has been associated with anticipatory responses that reflect cognitive expectations for pleasant/unpleasant experiences (appraising rewarding/aversive stimuli to generate emotional responses) (Ochsner et al., 2002; Ochsner and Gross, 2005). Specifically, increased mPFC activation has been noted during reappraisal and is associated with the suppressed subjective experience of negative emotions. Furthermore, an fMRI study revealed concurrent activation levels of the dorsomedial prefrontal cortex (dmPFC) with emotional valence when processing emotional stimuli: (i) activation was associated with positive valence, and (ii) deactivation was associated with negative valence (Heinzel et al., 2005). Similarly, emotional and non-emotional judgment task using the International Affective Pictures System (IAPS) demonstrated increased activation of the mPFC, specifically both ventromedial prefrontal cortex (vmPFC) and dmPFC during emotional judgment when compared with non-emotional judgment. However, an inverse relationship was observed in the lateral prefrontal cortex (VLPFC and DLPFC) during non-emotional judgment (Northoff et al., 2004). These findings suggested reciprocal interactions between cognitive and emotional processing between dorsal and lateral neural systems when processing emotional and cognitive tasking demands (Bartolic et al., 1999).

Other studies reported strong cognition-emotion interactions in the lateral prefrontal cortex with increased activity in the DLPFC, which plays a key role in top-down modulation of emotional processing (Northoff et al., 2004; Comte et al., 2014). This indicates increased attentional control of regulatory mechanisms that process emotional content. For instance, one study reported that cognitive task appeared to require active retention in WM, noting that the process was influenced by emotional stimuli when subjects were instructed to remember emotional valence information over a delay period (Perlstein et al., 2002). Their findings revealed increased activation in the right DLPFC in response to pleasant IAPS pictures, but with an opposite effect in response to unpleasant pictures (decreased activity in the right DLPFC). This could be interpreted as increased WM-related activity when processing positive emotional stimuli, thus leading to positive emotion maintenance of stimulus representation in WM. Furthermore, they observed that the DLPFC contributed to increased LTM performance linked to stronger item associations and greater organization of information in WM during pleasant compared to unpleasant emotion (Blumenfeld and Ranganath, 2006).

Another study investigated the PFC’s role in emotional mediation, reporting that the right VLPFC provided cognitive resources for both emotional reappraisal and learning processes via two separate subcortical pathways: (i) a path through NAc appeared to greater reappraisal success (suppress negative emotion) and (ii) another path through the ventral amygdala appeared to reduced reappraisal success (boost negative experience). This result indicates the VLPFC’s role in the regulation of emotional responses (reducing negative appraisal and generating positive appraisal) by retrieving appropriate information from memory (Wager et al., 2008). Certain characteristics of emotional content were found to mediate the encoding and retrieval of selective information by leading high levels of attention, distinctiveness, and information organization that enhanced recall for emotional aspects of complex events (Talmi, 2013). Hence, this direction of additional attention to emotional information appears to enhance LTM with the pronounced effects deriving from positive emotions compared with negative emotions. Effects of emotion on memory was also investigated using immediate (after 20 s) and delayed (after 50 min) testing paradigm, has shown that better recall for emotionally negative stimuli during immediate test compared to delayed test because of attentional allocation for encoding while the delayed test demonstrated that the role of amygdala in modulating memory consolidation of emotional stimuli. Because selective attention drives priority assignment for emotional material (Talmi et al., 2007). Meanwhile, the distinctiveness and organization of information can improve memory because unique attributes and inter-item elaboration during encoding serve as retrieval cues, which then lead to high possibilities for correct recall (Erk et al., 2003). Consistent findings were also reported by (Dolcos et al., 2004), who suggested an emotional mediation effect deriving from PFC activity in relation to cognitive functions such as strategic memory, semantic memory, and WM, which subsequently enhanced memory formation. **Table 1** summarizes cognitive-emotional functions associated with each sub-region of the PFC and corresponding Brodmann areas. Taken together, these findings indicate that the PFC is a key component in both cognitive and emotional processing for successful LTM formation and retrieval.

**Table 1:** The prefrontal cortex (PFC) sub-regions, corresponding Brodmann areas, and associated cognitive-emotional functions.

PFC region	BA	Functions	
		Cognitive	Emotional
aPFC	10	Engaged in higher-level cognitive functions (i.e., problem solving, planning and reasoning) and executive processes including WM (Koechlin et al., 1999).	Controls social-emotional interaction to coordinate rapid action selection processes, detection of emotional conflicts and inhibition of emotionally driven responses. Disruption leads to loss of control over automatic emotional tendencies and more errors in rule-driven responses (Volman et al., 2011).
		The pursuit of higher behavioral goals, with specialized roles in the explicit processing of internal mental states in WM, relational integration, and memory retrieval (Ramnani and Owen, 2004).	
DLPFC	9, 46	Left DLPFC manipulates information in WM while right DLPFC manipulates information in reasoning processes (Curtis and D’Esposito, 2003; Barbey et al., 2013).	Active maintenance of valence information in WM with increased WM-related activity in response to positive emotion (specifically in the right DLPFC) which leads to PFC-mediated cognitive functions in WM (i.e., increased cognitive flexibility and problem solving) (Ashby and Isen, 1999).
		Left DLPFC is associated with encoding and organization of material to be remembered; Right DLPFC is associated with memory retrieval (Dobbins et al., 2002).	Reward processing (Haber and Knutson, 2010). Emotion regulation (Ochsner and Gross, 2005).
VLPFC	44, 45, 47	Left VLPFC supports mnemonic control (i.e., task switching, WM and semantic retrieval), and supports access to stored conceptual representations (Badre and Wagner, 2007).	Emotion regulation (Opialla et al., 2015).
		Left VLPFC is involved in elaborative (semantic/phonological) encoding of information into episodic memory, the specification of retrieval cues and the maintenance of LTM retrieval (Poldrack et al., 1999; Wagner et al., 2001). Right VLPFC supports memory encoding and retrieval of visuospatial stimuli, action imitation and motor inhibition (Levy and Wagner, 2011).	Inhibition of distracting emotions (right VLPFC for inhibition of negative emotions) (Dolcos and McCarthy, 2006).
mPFC	25, 32	Learning, memory, and decision-making (Euston et al., 2012; Brod et al., 2013).	Dorsal-caudal mPFC involved in appraisal-expression of negative emotion; ventral-rostral PFC generates emotional regulation-responses (Etkin et al., 2011).
OFC	11, 12, 14	Decision making (Bechara et al., 2000).	Emotional processing and responses (Northoff et al., 2000), social and emotional judgment (Moll et al., 2002), facilitation of regret (Camille et al., 2004).
			Reward processing and reinforcement learning (Rolls, 2000).

*WM, working memory; PFC, prefrontal cortex; DLPFC, dorsolateral prefrontal cortex; VLPFC, ventrolateral prefrontal cortex; LTM, long-term memory; mPFC, medial prefrontal cortex.*

## Effects Deriving From Different Modalities of Emotional Stimuli on Learning and Memory

As discussed above, evidence indicates the neural mechanisms underlying the emotional processing of valence and arousal involve the amygdala and PFC, where the amygdala responds to emotionally arousing stimuli and the PFC responds to the emotional valence of non-arousing stimuli. We have thus far primarily discussed studies examining neural mechanisms underlying the processing of emotional images. However, recent neuroimaging studies have investigated a wider range of visual emotional stimuli. These include words (Sharot et al., 2004), pictures (Dolcos et al., 2005; Weymar et al., 2011), film clips (Cahill et al., 1996), and faces (González-Roldan et al., 2011), to investigate neural correlates of emotional processing and the impact of emotion on subsequent memory. These studies provided useful supplemental information for future research on emotional effects of educational multimedia content (combination of words and pictures), an increasingly widespread channel for teaching and learning.

An event-related fMRI study examined the neural correlates of responses to emotional pictures and words in which both were manipulated in terms of positive and negative valence, and where neutral emotional content served as a baseline (“conditioned stimuli”/no activating emotion with valence rating of 5 that spans between 1/negative valence-9/positive valence), even though all stimuli were consistent in terms of arousal levels (Kensinger and Schacter, 2006). Subjects were instructed to rate each stimulus as *animate* or *inanimate* and *common* or *uncommon*. The results revealed the activation of the amygdala in response to positive and negative valence (valence-independent) for pictures and words. A lateralization effect was observed in the amygdala when processing different emotional stimuli types. The left amygdala responded to words while either the right and/or bilateral amygdala activation regions responded to pictures. In addition, participants were more sensitive to emotional pictures than to emotional words. The mPFC responded more rigorously during the processing of positive than to that of negative stimuli, while the VLPFC responded more to negative stimuli. The researchers concluded that arousal-related responses occur in the amygdala, dmPFC, vmPFC, anterior temporal lobe and temporo-occipital junction, whereas valence-dependent responses were associated with the lateral PFC for negative stimuli and the mPFC for positive stimuli. The lateralization of the amygdala’s activation was consistent with that in other studies that also showed left-lateralized amygdala responses for words (Hamann and Mao, 2002) vs. right-lateralized amygdala responses for images (Pegna et al., 2005). However, a wide range of studies suggest that lateralization likely differs with *sex* (Hamann, 2005), *individual personality* (Hamann and Canli, 2004), *mood* (Rusting, 1998), *age* (Allard and Kensinger, 2014), *sleep* (Walker, 2009), *subject’s awareness of stimuli* (Morris et al., 1998), *stress* (Payne et al., 2007) and other variables. Hence, these factors should be considered in future studies.

Event-related potentials (ERPs) were used to investigate the modality effects deriving from emotional words and facial expressions as stimuli in healthy, native German speakers (Schacht and Sommer, 2009a). German verbs or pseudo-words associated with positive, negative or neutral emotions were used, in addition to happy vs. angry faces, as well as neutral and slightly distorted faces. The results revealed that negative posterior ERPs were evoked in the temporo-parieto-occipital regions, while enhanced positive ERPs were evoked in the fronto-central regions (positive verbs and happy faces) when compared with neutral and negative stimuli. These findings were in agreement with the previous findings (Schupp et al., 2003; Schacht and Sommer, 2009b). While the same neuronal mechanisms appear to be involved in response to both emotional stimuli types, latency differences were also reported with faster responses to facial stimuli than to words, likely owing to more direct access to neural circuits-approximately 130 ms for happy faces compared to 380 ms for positive verbs (Schacht and Sommer, 2009a). Moreover, augmented responses observed in the later positive complex (LPP), i.e., larger late positive waves in response to emotional verbs (both positive and negative) and angry faces, all associated with the increased motivational significance of emotional stimuli (Schupp et al., 2000) and increased selective attention to pictures (Kok, 2000).

Khairudin et al. (2011) investigated effects of emotional content on explicit memory with two standardized stimuli: emotional words from the Affective Norms for English Words (ANEW) and emotional pictures from the IAPS. All stimuli were categorized as positive, negative or neutral, and displayed in two different trials. Results revealed that better memory for emotional images than for emotional words. Moreover, a recognition test demonstrated that positive emotional content was remembered better than negative emotional content. Researchers concluded that emotional valence significantly impacts memory and that negative valence suppressed the explicit memory. Another study by Khairudin et al. (2012) investigated the effects of emotional content on explicit verbal memory by assessing recall and recognition for emotionally positive, negative and neutral words. The results revealed that emotion substantially influences memory performance and that both positive and negative words were remembered more effectively than neutral words. Moreover, emotional words were remembered better in recognition vs. recall test.

Another group studied the impacts of emotion on memory using emotional film clips that varied in emotion with neutral, positive, negative and arousing contents (Anderson and Shimamura, 2005). A subjective experiment for word recall and context recognition revealed that memory, for words associated with emotionally negative film clips, was lower than emotionally neutral, positive and arousing films. Moreover, emotionally arousing film clips were associated with enhanced context recognition memory but not during a free word recall test. Therefore, clarifying whether emotional stimuli enhance recognition memory or recall memory requires further investigation, as it appears that emotional information was better remembered for recognition compared to recall. In brief, greater attentional resource toward emotional pictures with large late positive waves of LPP in the posterior region, the amygdala responds to emotional stimuli (both words and pictures) independent on its valence, leading to enhanced memory. **Table 2** summarizes studies on the brain regions that respond to standardized stimuli as cited above, and also for pictures of emotional facial expression or Pictures of Facial Affect (POFA), Affective Norms for English Words (ANEW) for emotional words, as well as for the International Affective Digitized Sound System (IDAS) for emotional sounds.

**Table 2:** Comparison of different emotional stimulus categories.

Study	Stimulus types	Emotion categories	Investigation	Brain imaging modality	Brain regions of interest	Findings	Subjects	Status	Age
Kensinger and Schacter, 2006	Pictures (IAPS) and words (ANEW)	Positive, negative, and neutral	Brain responses to emotionally positive, negative, and arousing words	Event-related fMRI	Amygdala, PFC, anterior temporal lobe, and temporooccipital junction	∙ Amygdala, dmPFC, and vmPFC responded equally to both pictures and words regardless of valence. ∙ mPFC was more activated for positive content. ∙ VLPFC was more activated for negative content. ∙ Greater sensitivity for emotional pictures than words.	21 adults (10 Female, 11 Male)	Healthy	18–35 years
Hamann and Mao, 2002	Words (ANEW)	High-arousal positive, high-arousal negative, and neutral	Brain responses to positive and negative emotionally arousing words	Event-related fMRI	Amygdala, vmPFC	∙ Left amygdala activated for both positive and negative words. ∙ No activation observed in the vmPFC in response to positive or negative words.	14 adults (All)	Healthy	20–31 years
Pegna et al., 2005	Faces	Positive, negative, and neutral	Responses to emotional face expression without primary visual areas	Event-related fMRI	Amygdala	∙ Right amygdala activated for all emotional faces (anger, happiness, and fear).	1 Male	Blind sight patient	52 years
Canli et al., 2000	Pictures (IAPS)	Negative and neutral	Amygdala response to emotional experience during study and LTM	Event-related fMRI	Amygdala	∙ Left amygdala activation during encoding was a predictor of subsequent recognition memory for pictures with high emotional intensity ratings.	10 Female	Healthy	–
Krause et al., 2000	Film clips	Aggressive, sad, and neutral	Responses of EEG frequency bands on the emotional film content	EEG	Occipital (Posterior), central and frontal (anterior)	∙ EEG theta (4–6 Hz) was more synchronized in occipital and frontal regions for the aggressive films compared with neutral films. ∙ EEG theta (4–6 Hz) respond specifically to visual emotional stimulus. ∙ EEG alpha is associated with attention and habituation.	18 adults (All Female)	Healthy	20–33 years
Cuthbert et al., 2000	Pictures (IAPS)	Pleasant, neutral, and unpleasant	Brain responses to emotional pictures	ERP	Midline (Fz, Cz, and Pz)	∙ More positivity for pleasant and unpleasant pictures than neutral pictures in the posterior regions. ∙ An indication of selective emotional processing (resulted from the motivational relevance of emotional pictures compared to neutral ones).	14 Female	–	18–24 years
Hinojosa et al., 2009	Words (Spanish nouns) Pictures (IAPS)	Negative, positive, neutral, and relaxing	Processing of emotional information in words and pictures	ERP	Frontal and parieto-occipital Centro-parietal and frontal regions	∙ Both emotional words and pictures were associated with an early posterior negativity and LPC. ∙ Emotional pictures elicited greater amplitude of early posterior negativity after stimulus presentation at the frontal and parieto-occipital regions. ∙ Positive pictures were associated with enhanced early posterior negativity amplitude in the right parieto-occipital regions. ∙ An arousal-dependent effect was observed in the left parieto-occipital regions for both positive and negative stimuli.	21 volunteers (19 Female, 2 Male) 28 volunteers (21 Female, 7 Male)	Healthy Healthy	19–27 years 19–29 years
Holmes et al., 2003	Facial expression (POFA)	Fearful vs. neutral	Spatial attention effects on emotional face processing.	ERP	Frontal, central and posterior regions	Faces enhanced N170 amplitude reflecting that spatial attention modulates face encoding at lateral posterior electrodes. However, N170 was insensitive to emotional expression.	20 subjects (11 Female, 7 Male, 2 excluded due to excess artifacts)	Healthy	18–32 years
Bayer et al., 2010	Sentence	Negative/high arousal and Neutral/ low arousal	Impact of emotional verb processing in short sentences (Reading)	ERP	Centro-parietal regions	Effect on LPC of negative and high-arousal words, while LPC was not affected by arousal-related words alone. Reported the importance of valence and arousal in emotion-related ERP effects.	21 participants (11 Female, 10 Male)	Healthy	–
Plichta et al., 2011	Sound (IADS)	Pleasant, unpleasant, and neutral	Auditory cortex response to emotional stimuli	fNIRS	Auditory cortex	Both pleasant and unpleasant sounds led to greater activation in the left and right auditory cortex compared with neutral sound.	17 participants (10 Female, 7 Male)	Healthy	–

*IAPS, International Affective Picture System; ANEW, Affective Norms for English Words; POFA, Pictures of Facial Affect; IADS, International Affective Digitized Sound System; ERP, event-related potential; fMRI, functional magnetic resonance imaging; fNIRS, functional near-infrared spectroscopy; PFC, prefrontal cortex; vmPFC, ventromedial prefrontal cortex; dmPFC, dorsomedial prefrontal cortex; mPFC, medial prefrontal cortex; VLPFC, ventrolateral prefrontal cortex; LPP, late positive potential; LPC, late positive complex; LTM, long-term memory; EEG, electroencephalography.*

## Neuroimaging Techniques for the Investigation of Emotional-Cognitive Interactions

The brain regions associated with cognitive-emotional interactions can be studied with different functional neuroimaging techniques (fMRI, PET, and fNIRS) to examine hemodynamic responses (indirect measurement). EEG is used to measure brain electrical dynamics (direct measurement) associated with responses to cognitive and emotional tasks. Each technique has particular strengths and weaknesses, as described below.

### Functional Magnetic Resonance Imaging (fMRI)

Functional magnetic resonance imaging is a widely used functional neuroimaging tool for mapping of brain activation as it provides a high spatial resolution (a few millimeters). fMRI is an indirect measure of hemodynamic response by measuring changes in local ratios of oxy-hemoglobin vs. deoxy-hemoglobin, typically known as a blood oxygenation level dependent (BOLD) signal (Cabeza and Nyberg, 2000). Dolcos et al. (2005) examined the effects of emotional content on memory enhancement during retrieval process using event-related fMRI to measure retrieval-related activity after a retention interval of 1 year. The researchers concluded that successful retrieval of emotional pictures involved greater activation of the amygdala as well as the entorhinal cortex and hippocampus than that of neutral pictures. Both the amygdala and hippocampus were rigorously activated during recollection compared to familiarity recognition, whereas no differences were found in the entorhinal cortex for either recollection or familiarity recognition. Moreover, a study investigates motivation effect (low vs. high monetary reward) on episodic retrieval by manipulating task difficulty, fMRI data reports that increased activation in the substantia nigra/VTA, MTL, dmPFC, and DLPFC when successful memory retrieval with high difficulty than with low difficulty. Moreover, reward-related of functional connectivities between the (i) SN/VTA–MTL and (ii) SN/VTA–dmPFC appear to increases significantly with increases retrieval accuracy and subjective motivation. Thus, Shigemune et al. (2017) suggest that reward/motivation-related memory enhancement modulated by networking between the SN/VTA (reward-related), dmPFC (motivation-related) and MTL (memory-related) network as well as DLPFC (cognitive controls) with high task difficulty.

Taken together, these findings indicate that the amygdala and MTL have important roles in the recollection of emotional and motivational memory. Another fMRI study reported that greater success for emotional retrieval (emotional *hits* > *misses*) was associated with neural activation of the bilateral amygdala, hippocampus, and parahippocampus, whereas a higher success rate for neutral retrieval is associated with a greater activity in right posterior parahippocampus regions (Shafer and Dolcos, 2014). Hence, fMRI has clearly revealed interactions between cognitive and emotional neural networks during information processing, particularly in response to emotion-related content. Such interactions appear to modulate memory consolidation while also mediating encoding and retrieval processes that underlie successful LTM formation and memory recall. More specifically, it appears that amygdala activation modulates both the hippocampus and visual cortex during visual perception and enhances the selection and organization of salient information via the “bottom-up” approach to higher cognitive functions directed at awareness. Although fMRI is widely used, it poses several limitations such as poor temporal resolution, expensive setup costs, plus the difficulty of having a subject hold still during the procedure in an electromagnetically shielded room (immobility). Furthermore, fMRI is slightly more metabolically sluggish, as BOLD signal exhibits an initial dip, where the increase of subsequent signal is delayed by 2–3 s and it takes approximately 6–12 s to reach to a peak value that reflects the neural responses elicited by a stimulus (Logothetis et al., 2001). This means that fMRI having a coarse temporal resolution (several seconds) when compared with electrophysiological techniques (a few milliseconds) and also not a great technique for visualizing subcortical regions (mesencephalon and brainstem) due to metabolically sluggish compared to PET.

### Positron Emission Tomography (PET)

Positron emission tomography is another functional neuroimaging tool that maps CNS physiology and neural activation by measuring glucose metabolism or regional cerebral blood flow (rCBF). PET uses positron-emitting radionuclides such as ^18^F-fluorodeoxyglucose (FDG) and positron-emitting-oxygen isotope tagged with water ([^15^O] H_2_O), etc. This technique identifies different neural networks involving pleasant, unpleasant and neutral emotions (Lane et al., 1997). It thus far appears that increased rCBF in the mPFC, thalamus, hypothalamus, and midbrain associated with pleasant and unpleasant emotional processing, while unpleasant emotions are more specifically associated with the bilateral OTC, cerebellum, left parahippocampal gyrus, hippocampus, and amygdala; moreover, the caudate nucleus is associated with pleasant emotions.

Using PET scanning demonstrated that emotional information enhances visual memory recognition via interactions between perception and memory systems, specifically with greater activation of the lingual gyrus for visual stimuli (Taylor et al., 1998). The results also showed that strong negative emotional valence appeared to enhance the processing of early sensory input. Moreover, differences in neural activation appeared in the left amygdaloid complex (AC) during encoding, while the right PFC and mPFC responded during recognition memory. Similarly, Tataranni et al. (1999) identified CNS regions associated with appetitive states (hunger and satiation) (Tataranni et al., 1999). Hunger stimulated increased rCBF uptake in multiple regions including the hypothalamus, insular cortex, limbic and paralimbic regions (anterior cingulate cortex, parahippocampal and hippocampal formation, the anterior temporal and posterior orbitofrontal cortex), as well as the thalamus, caudate, precuneus, putamen, and cerebellum. Satiation was associated with increased rCBF uptake in the bilateral vmPFC, the DLPFC, and the inferior parietal lobule. These results imply that (i) subcortical regions associated with emotion/motivation involved in hunger that signals distressing feeling (discomfort, pain and anxiety) for the regulation of food intake; and (ii) the PFC associated with inhibition of inappropriate behavioral response involved in satiation that signals excessive food consumption for a termination of meal.

In a study of emotional self-generation using PET noted that the insular cortex, secondary somatosensory cortex, and hypothalamus, as well as the cingulate cortex and nuclei in the brainstem’s tegmentum, including PAG, parabrachial nucleus, and substantia nigra maintained current homeostasis by generating regulatory signals (Damasio et al., 2000). PET scanning has also been used for neuroanatomical mapping of emotions (Davidson and Irwin, 1999), emotional processing (Choudhary et al., 2015), and cognitive functions (Cabeza and Nyberg, 2000). Although PET scanning has a relatively good spatial resolution for both the brain and bodily functions, it is costly and yields lower temporal resolution than does EEG and is invasive as opposed to fMRI. Moreover, PET tends to show better activation of more ancient brain regions in the mesencephalon and brainstem when compared to fMRI. Hence, it is generally reserved for the clinical diagnoses of cancers, neurological diseases processes (e.g., epilepsy and Alzheimer’s disease), and heart diseases.

### Electroencephalography (EEG)

Electroencephalography obtains high temporal resolution in milliseconds, portable, less expensive, and non-invasive techniques by attaching scalp electrodes to record brain electrical activity. Moreover, numerous studies reported that EEG is useful in mapping CNS cognitive and emotional processing. The technique offers a comprehensive range of feature extraction and analysis methods, including power spectral analysis, EEG coherence, phase delay, and cross-power analysis. One study examined changes in EEG oscillations in the amygdala during the consolidation of emotionally aroused memory processing that exhibited theta (4–8 Hz) activity (Paré et al., 2002), indicating the facilitation of memory consolidation, improved retention of emotional content, and enhanced memory recall. This finding was later supported by the revelation of increased theta activity in the right frontal (Friese et al., 2013) and right temporal cortices (Sederberg et al., 2003) and consequently associated with the successful encoding of new information. Another study (Buzsáki, 2002) revealed that theta oscillations were positively related to the activation of the hippocampus represent the active brain state during sensory, motor and memory-related processing. The theta waves are generated through an interaction between the entorhinal cortex, the Schaffer collateral (CA3 region) and the pyramidal cell dendrites (both CA3 and CA1 regions) that result in a synaptic modification underlie learning and memory. Thus, theta oscillation is thought to be associated with the encoding of new memories.

Electroencephalography studies have also revealed alpha asymmetry over prefrontal regions during withdrawal/avoidance processing. Electrophysiological responses showed increased alpha-band activity in the right vs. left PFC when subjects viewed film clips with withdraw-related negative emotional content (Papousek et al., 2014). EEG alpha wave power is inversely related to cortical activity, that is, a lower alpha power associated with higher activity (inhibition). Because of this cortical inhibition consideration, researcher (Davidson, 1988) introduces *frontal asymmetry index* by computing a ratio of differences in log-transformation power values between the left frontal and right frontal alpha power or 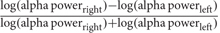, and the selection of electrodes is the homologous pairs (F3-F4/F7-F8). Thus, positive value indicates left frontal activity that associated with positive emotion/approach motivation and negative value indicates right frontal activity that associated with negative emotion/withdrawal motivation. In other words, greater right alpha power (right frontal activation ↓) than left alpha power (left frontal activation ↑) results in left frontal activity and vice versa. Another study reported greater alpha activity in the left frontal region (less left frontal alpha power) was associated with *approach* motivation, while the greater alpha activity in right frontal (less right frontal alpha power) was associated with *withdrawal* motivation (Harmon-Jones et al., 2010). Hence, these findings indicate that EEG alpha asymmetry may be used to assess “approach” (positive valence) vs. “withdrawal” (negative valence) motivational processes and/or emotional responses during learning. However, anger is associated with approach motivation (but negative valence) state appeared to have a greater left frontal activity compared with positively valenced approach motivation (happiness) (Harmon-Jones and Gable, 2017). Because of inconsistencies of *valence hypothesis* and *motivational direction hypothesis*, a study proposed *asymmetry inhibition model* of alpha activity with two mechanisms of inhibitory executive control to regulate emotional distraction processing: (i) left PFC inhibits processing of negative/withdrawal-related distractors and (ii) right PFC inhibits processing of positive/approach-related distractors (Grimshaw and Carmel, 2014). Because of there is a strong inverse association between alpha and the fronto-parietal network, which increase of alpha activity associated with a decrease fronto-parietal activity that reflects the executive control mechanism inhibits interference from irrelevant emotional distractors.

Increased gamma oscillation in the neocortex and right amygdala have been reported in response to emotionally arousing pictures during learning and memory tasks undertaken by 148 right-handed female participants (Headley and Paré, 2013). A more detailed study by Müller et al. (1999) reported increased gamma potentials in the left frontal and temporal regions in response to images having a negative valence, whereas increased gamma-bands in the right frontal regions were observed in responses to images with positive valence for 11 right-handed male participants. During an emotionally positive experience, another study reported significantly increased EEG theta-alpha coherence between prefrontal and posterior parietal regions (Aftanas and Golocheikine, 2001). They concluded the change was associated with heightened attention in association with improved performance in memory and emotional processing. Thus, we have a number of EEG investigations of left and right hemispheric activity while processing positive (pleasant) and negative (unpleasant) stimuli that revealed differences in regional electrophysiological activation. Nonetheless, EEG exhibits a relatively poor spatial resolution approximately 5 to 9 cm compared with fMRI and PET (Babiloni et al., 2001). Thus, scalp EEG unable to measure activation much below cortex owing to the distortion of scalp potentials where different volume conduction effects of the cortex, dura mater, skull, and scalp resulting in imprecise localization of the electromagnetic field patterns associated with neural current flow. Subsequent studies have demonstrated that the EEG spatial resolution can be improved using high-resolution EEG (high-density electrode arrays to increase spatial sampling) with surface Laplacian estimation and cortical imaging (details discussion of this area is beyond the scope of this review, see (Nunez et al., 1994) for theoretical and experimental study) or integrating multiple imaging modalities that provide complement information, for instance EEG-fMRI and EEG-fNIRS (Dale and Halgren, 2001).

### Functional Near-Infrared Spectroscopy (fNIRS)

Functional near-infrared spectroscopy is an emerging and relatively low-cost imaging technique that is also portable and non-invasive. It can be used to map the hemodynamic responses associated with brain activation. This technology measures cerebral changes in the concentration of oxygenated hemoglobin (oxy-Hb) vs. deoxygenated hemoglobin (deoxy-Hb) using optodes (light emitters and detectors) placed on the scalp (Villringer et al., 1993). It is limited to visualizations of cortical activity compared to the subcortical regions, and findings only imply increased brain activity associated with increased glucose and oxygen consumption. Elevations in cerebral blood flow and oxygen delivery exceed quo oxygen consumption, thereby enabling changes in local cerebral blood oxygenation to be measured by optic penetration.

The number of studies that have implemented this investigative technique are associated with task performance (Villringer et al., 1993), including *exercise* (Perrey, 2008), *cognitive workload* (Durantin et al., 2014), *psychiatric disorders* (Ehlis et al., 2014), *emotional processing* (Bendall et al., 2016), and *aging* (Hock et al., 1995). One study used fNIRS to examine the relationship between subjective happiness and emotional changes (Oonishi et al., 2014). The results revealed that the level of subjective happiness influenced the pattern of left-right PFC activation during the emotion-related task, showing increased oxy-Hb in the left PFC when viewing pleasant pictures, and increased oxy-Hb in the right PFC when viewing unpleasant pictures. Viewing unpleasant emotional stimuli accompanied increased in oxy-Hb levels in the bilateral VLPFC while also activating several regions in both the right VLPFC (BA45/47) and left VLPFC (BA10/45/46/47). However, another fNIRS study reported that viewing pleasant emotional stimuli was associated with decreased oxy-Hb in the left DLPFC (BA46/10) when affective images were presented for 6 s (Hoshi et al., 2011). Thus, this study found an opposite pattern indicating left hemisphere involvement in positive/approach processing and right hemisphere involvement in negative/withdrawal processing (Davidson, 1992; Davidson and Irwin, 1999). This inconsistent finding of frontal hemispheric asymmetric might result from the comparison of state-related changes rather than baseline levels of asymmetric. Thus, several issues should take into consideration: (i) *methodological* issues to assess hemispheric asymmetry, including requires repeat measures of anterior asymmetry for at least two sessions, stimulus content should comprise both positive valence and negative valence while maintaining at a similar level of arousal and with a baseline resting condition, appropriate selection of reference electrode and individual differences, etc; and (ii) *conceptual* issues is related to the fact that prefrontal cortex is an anatomically and functionally heterogeneous and complex region interacts with other cortical and subcortical structures during emotional processing (Davidson, 2004). Another fNIRS study examined the relationship between PFC function and cognitive control of emotion (Ozawa et al., 2014). This was done by presenting emotional IAPS pictures for 5.2 s, followed by the *n*-back task. The results revealed a significantly greater increase in oxy-HB in the mPFC and left superior frontal gyrus in response to negative pictures compared with neutral pictures. Meanwhile, no significant hemodynamic changes were observed during image presentation and the *n*-back task, indicating the need for further investigation.

## Factors Affecting the Effect of Emotion on Learning and Memory

The preceding section described neuroimaging techniques used to examine brain responses to emotional stimuli during WM processing leading to LTM. This section presents six key factors that are recommended for consideration in the experimental design and appropriate protocol.

### Individual Differences

A number of studies have reported numerous influences in addition to a range of individual differences in emotional processing. These include *personality traits* (Montag and Panksepp, 2017), *intellectual ability* (Brackett et al., 2004), and *sex* (Cahill, 2003). Moreover, sex hormones and personality traits (e.g., extraversion and neuroticism) appear to influence individual responses to emotional stimuli as well as modulate emotional processing. Appropriate screening with psychological testing as well as balancing experimental cohorts in terms of sex can help reduce spurious results owing to individual differences.

### Age-Related Differences

Studies have also shown that older adults are associated with the greater familiarity with psychological stress and emotional experiences, thus causing *positivity biases* in emotional processing and better emotional control than in younger adults (Urry and Gross, 2010; Allard and Kensinger, 2014). Consequently, the age of participants in a sample population should be considered for both cognitive and emotional studies.

### Emotional Stimulus Selection

The selection of emotional stimuli for experimental studies is generally divided into two streams: (1) discrete emotional, and (2) dimensional emotions of valence, arousal, dominance and familiarity (Russell, 1980; Barrett, 1998). The latter include pictures from the IAPS database and words from the ANEW database, which are both available for non-commercial research. Appropriate selection of emotional stimuli is another important consideration that ensures experimental tasks are suitable for the investigation of emotional processing in learning and memory. Furthermore, the type of stimulus determines stimulus presentation duration, especially for experimental tasks involving the induction of emotions.

### Self-assessment Techniques

There are numerous self-assessment techniques used to measure individual emotional states (Bradley and Lang, 1994). The most widely used techniques are the Self-Assessment Manikin (SAM), the Semantic Differential (SD) scale, and the Likert scale. The SAM is a non-verbal pictorial assessment technique directly measures emotional responses to emotional stimuli for valence, arousal, and dominance. The SD scale consists of a set of bipolar adjective pairs for the subjective rating of image stimuli. The Likert’s “*x*-point” scale allows participants to rate their own emotional responses. If a study does not seek to assess distinct emotional states but rather involves the assessment of two primary dimensions of emotion (positive and negative valence), then the Positive and Negative Affect Schedule (PANAS) is a recommended method (Watson et al., 1988). Thus, selection of the most appropriate self-assessment technique is an important part of the experimental design but can also become an overwhelming task.

### Selection of Brain Imaging Techniques

As mentioned above, the two major types of brain imaging techniques EEG (direct) and fMRI/PET/fNIRS (indirect) have respective advantages and disadvantages. To overcome these limitations, simultaneous or combined dual-modality imaging (EEG-fMRI or EEG-fNIRS) can now be implemented for complementary data collection. Although functional neuroimaging works to identify the neural correlates of emotional states, technologies such as deep brain stimulation (DBS) and connectivity maps might provide new opportunities to seek understanding of emotions and its corresponding psychological responses.

### Neurocognitive Research Design

The neuroscience of cognition and emotion requires appropriate task designs to accomplish specific study objectives (Amin and Malik, 2013). Environmental factors, ethical issues, memory paradigms, cognitive task difficulty, and emotional induction task intensity must be considered for this.

Numerous neuroimaging studies cited thus far have indicated that emotions influence memory processes, to include memory encoding, memory consolidation, and memory retrieval. Emotional attentional and motivational components might explain why emotional content exhibits privileged information processing. Emotion has a “pop-out” effect that increases attention and promotes bottom-up instinctual impact that enhances awareness. Significant emotional modulation affects memory consolidation in the amygdala, and emotional content also appears to mediate memory encoding and retrieval in the PFC, leading to slow rates of memory lapse accompanied by the accurate recall. Moreover, cognitive and emotional interactions also appear to modulate additional memory-related CNS regions, such as the frontal, posterior parietal and visual cortices. The latter are involved in attentional control, association information, and the processing of visual information, respectively. Therefore, higher-level cognitive functions such as learning and memory, appear to be generally guided by emotion, as outlined in the Panksepp’s framework of brain processing (Panksepp, 1998).

Neuroimaging findings also indicate the involvement of the PFC in emotional processing by indirectly influencing WM and semantic memory (Kensinger and Corkin, 2003). This is reflected by the involvement of the DLPFC in WM and the role played by VLPFC in semantic processing, both of which have been found to enhance or impair semantic encoding task performance when emotion is involved. Various parts of the lateral PFC (ventrolateral, dorsolateral and medial prefrontal cortical regions) are suspected of having key roles that support memory retrieval (Simons and Spiers, 2003). All of these findings suggest that PFC-MTL interactions underlie effective semantic memory encoding and thus strategically mediate information processing with increased transfer to the hippocampus, consequently enhancing memory retrieval. Accordingly, learning strategies that emphasize emotional factors are more likely to result in long-term knowledge retention. This consideration is potentially useful in the design of educational materials for academic settings and informed intelligent tutoring systems.

Based on numerous previous findings, future research might take emotional factors more seriously and more explicitly in terms of their potential impact on learning. By monitoring the emotional state of students, the utilization of scientifically derived knowledge of stimulus selection can be particularly useful in the identification of emotional states that advance learning performance and outcomes in educational settings. Moreover, functional neuroimaging investigations now include single and/or combined modalities that obtain complementary datasets that inform a more comprehensive overview of neuronal activity in its entirety. For example, curiosity and motivation promote learning, as it appears cognitive network become energized by the mesolimbic-mesocortical dopamine system (generalized motivational arousal/SEEKING system). In addition, the identification of emotional impact on learning and memory potentially has direct implications for healthy individuals as well as patients with psychiatric disorders such as depression, anxiety, schizophrenia, autism, mania, obsessive-compulsive disorder and post-traumatic stress disorder (PTSD) (Panksepp, 2011a). To emphasize, depression and anxiety are the two most commonly diagnosed psychiatric disorders associated with learning/memory impairment and pose negative consequences that (i) limit the total amount of information that can otherwise be learned, and (ii) inhibit immediate recall as well as memory retention and retrieval of newly learned information. Depression and anxiety are also associated with negative emotions such as hopelessness, anxiety, apathy, attention deficit, lack of motivation, and motor and mental insufficiencies. Likewise, neuroscience studies report that decreased activation of the dorsal limbic (the anterior and posterior cingulate) as well as in the prefrontal, premotor and parietal cortices causes attentional disturbance, while increased neural activation in the ventral paralimbic region (the subgenual cingulate, anterior insula, hypothalamus and caudate) is associated with emotional and motivational disorders (Mayberg, 1997).

## Concluding Remarks, Open Questions, and Future Directions

Substantial evidence has established that emotional events are remembered more clearly, accurately and for longer periods of time than are neutral events. Emotional memory enhancement appears to involve the integration of cognitive and emotional neural networks, in which activation of the amygdala enhances the processing of emotionally arousing stimuli while also modulating enhanced memory consolidation along with other memory-related brain regions, particularly the amygdala, hippocampus, MTL, as well as the visual, frontal and parietal cortices. Similarly, activation of the PFC enhances cognitive functions, such as strategic and semantic processing that affect WM and also promote the establishment of LTM. Previous studies have primarily used standardized emotional visual, or auditory stimuli such as pictures, words, facial expression, and film clips, often based on the IAPS, ANEW, and POFA databases for emotional pictures, words and facial expressions, respectively. Further studies have typically focused on the way individuals memorize (intentional or incidental episodic memory paradigm) emotional stimuli in controlled laboratory settings. To our knowledge, there are few objective studies that employed brain-mapping techniques to examine semantic memory of learning materials (using subject matter) in the education context. Furthermore, influences derived from emotional factors in human learning and memory remains unclear as to whether positive emotions facilitate learning or negative emotions impair learning and vice versa. Thus, several remaining questions should be addressed in future studies, including (i) the impact of emotion on semantic knowledge encoding and retrieval, (ii) psychological and physiological changes associated with semantic learning and memory, and (iii) the development of methods that incorporate emotional and motivational aspects that improve educational praxes, outcomes, and instruments. The results of studies on emotion using educational learning materials can indeed provide beneficial information for informed designs of new educational courses that obtain more effective teaching and help establish better informed learning environments. Hence, to understand how emotion influence learning and memory requires understanding of an evolutionary consideration of the nested hierarchies of CNS emotional-affective processes as well as a large-scale network, including the midbrain’s PAG and VTA, basal ganglia (amygdala and NAc), and insula, as well as diencephalon (the cingulate and medial frontal cortices through the lateral and medial hypothalamus and medial thalamus) together with the MTL, including the hippocampus as well as the entorhinal cortex, perirhinal cortex, and parahippocampal cortices that responsible for declarative memories. Moreover, the SEEKING system generates positive subjective emotional states-positive expectancy, enthusiastic exploration, and hopefulness, apparently, initiates learning and memory in the brain. All cognitive activity is motivated from ‘underneath’ by basic emotional and homeostatic needs (motivational drives) that explore environmental events for survival while facilitating secondary processes of learning and memory.

## Author Contributions

CMT drafted this manuscript. CMT, HUA, MNMS, and ASM revised this draft. All authors reviewed and approved this manuscript.

## Conflict of Interest Statement

The authors declare that the research was conducted in the absence of any commercial or financial relationships that could be construed as a potential conflict of interest.
